# The Parasexual Cycle in Candida albicans Provides an Alternative Pathway to Meiosis for the Formation of Recombinant Strains

**DOI:** 10.1371/journal.pbio.0060110

**Published:** 2008-05-06

**Authors:** Anja Forche, Kevin Alby, Dana Schaefer, Alexander D Johnson, Judith Berman, Richard J Bennett

**Affiliations:** 1 Department of Genetics, Cell Biology and Development, University of Minnesota, Minneapolis, Minnesota, United States of America; 2 Department of Molecular Microbiology and Immunology, Brown University, Providence, Rhode Island, United States of America; 3 Departments of Biochemistry and Biophysics and Microbiology and Immunology, University of California San Francisco, San Francisco, California, United States of America; Duke University, United States of America

## Abstract

Candida albicans has an elaborate, yet efficient, mating system that promotes conjugation between diploid **a** and α strains. The product of mating is a tetraploid **a**/α cell that must undergo a reductional division to return to the diploid state. Despite the presence of several “meiosis-specific” genes in the C. albicans genome, a meiotic program has not been observed. Instead, tetraploid products of mating can be induced to undergo efficient, random chromosome loss, often producing strains that are diploid, or close to diploid, in ploidy. Using SNP and comparative genome hybridization arrays we have now analyzed the genotypes of products from the C. albicans parasexual cycle. We show that the parasexual cycle generates progeny strains with shuffled combinations of the eight C. albicans chromosomes. In addition, several isolates had undergone extensive genetic recombination between homologous chromosomes, including multiple gene conversion events. Progeny strains exhibited altered colony morphologies on laboratory media, demonstrating that the parasexual cycle generates phenotypic variants of C. albicans. In several fungi, including Saccharomyces cerevisiae and *Schizosaccharomyces pombe*, the conserved Spo11 protein is integral to meiotic recombination, where it is required for the formation of DNA double-strand breaks. We show that deletion of *SPO11* prevented genetic recombination between homologous chromosomes during the C. albicans parasexual cycle. These findings suggest that at least one meiosis-specific gene has been re-programmed to mediate genetic recombination during the alternative parasexual life cycle of C. albicans. We discuss, in light of the long association of C. albicans with warm-blooded animals, the potential advantages of a parasexual cycle over a conventional sexual cycle.

## Author Summary

Candida albicans is an important human fungal pathogen that has an unconventional sexual cycle. Efficient mating requires that diploid cells of opposite mating type first switch from the more common “white” phase to the “opaque” phase and then undergo cell fusion. The resulting tetraploid strains can return to the diploid state via a non-meiotic parasexual program of concerted chromosome loss. We used SNP and comparative genome hybridization to analyze the progeny resulting from this parasexual cycle and found a range of genetically diverse strains with altered phenotypes. In addition, in a subset of these strains, genetic recombination was found to have taken place between homologous chromosomes. This recombination was dependent on Spo11, a conserved protein required for the introduction of DNA double-strand breaks in the chromosomes of eukaryotes that undergo conventional meiosis. Thus, Spo11 is required for genetic recombination and the generation of increased genetic diversity during the C. albicans parasexual cycle.

## Abstract 3

The opportunistic pathogen *Candida albicans* has a cryptic parasexual mating cycle involving epigenetic switching and chromosome loss. This study demonstrates that genetic recombination occurs during the mating cycle and that this requires the activity of Spo11, a protein necessary for meiotic recombination in other eukaryotes.

## Introduction

In most sexually reproducing eukaryotes, meiosis is used to precisely halve the DNA content in the cell, often for the formation of haploid gametes from diploid precursor cells. This specialized form of cell division involves one round of DNA replication followed by two successive rounds of DNA division. Each round of DNA division is unique. During the first meiotic division (meiosis I) extensive DNA recombination takes place between maternal and paternal homologous chromosomes, which then are segregated from one another. The second round of DNA division (meiosis II) more closely resembles normal mitotic DNA division, in which sister chromatids are segregated to opposite poles. In the case of spores in fungi and spermatozoa in animals, all four haploid nuclei form four different haploid cells, while in the female meioses of animals only one haploid nucleus survives and forms the mature oocyte.

The meiotic process has been studied extensively in the model fungi Saccharomyces cerevisiae and Schizosaccharomyces pombe. In S. cerevisiae, mating of haploid *MAT**a*** and *MATα* cells normally generates a stable diploid **a**/α cell that replicates mitotically until subsequently induced to undergo meiosis under conditions of limiting nitrogen availability and the presence of a non-fermentable carbon source [1]. In S. pombe, mating also occurs between haploid cells but the diploid state is often transient, immediately undergoing meiosis to regenerate the haploid form. The sexual program in S. pombe is again controlled by nutritional cues, as mating and meiosis normally occur only under starvation conditions [2]. In both S. cerevisiae and S. pombe, meiosis generates four recombinant haploid spores held together in an ascus.

While S. cerevisiae and S. pombe are rarely pathogenic in humans, the related ascomycete C. albicans is an opportunistic pathogen capable of causing both debilitating mucosal infections and potentially life-threatening systemic infections [3]. C. albicans is normally a harmless commensal fungus, existing in the gastrointestinal tract of at least 70% of the healthy population [4]. However, C. albicans is also the most commonly isolated fungal pathogen, particularly targeting individuals with compromised immune systems and leading to death in up to 50% of patients with bloodstream infections [5–7].

Until recently, C. albicans was thought to be asexual, existing only as an obligate diploid organism and thus classified amongst the Fungi imperfecti [8]. However, a robust mating system has now been uncovered in this organism, in which mating occurs between diploid mating type-like (*MTL*) **a** and α strains to generate an **a**/α tetraploid strain. Mating occurs both under laboratory conditions and in different in vivo niches in a mammalian host [9–12]. Population studies of clinical isolates are also consistent with C. albicans strains undergoing genetic exchange in their natural environment, albeit at a limited rate [13].

While an efficient mating apparatus has now been identified in C. albicans, the mating cycle differs in several important respects from that of S. cerevisiae and other fungi. For example, mating in C. albicans is regulated by phenotypic switching; *MTL* homozygous C. albicans cells can reversibly switch between two heritable states termed white and opaque, and only the opaque form is competent for efficient mating [14]. This unusual mode of mating regulation is so far unique to C. albicans (and the very closely related yeast, Candida dubliniensis [15]) making it likely that this adaptation has evolved to regulate mating of C. albicans strains in their natural environment—that of a warm-blooded host. Completion of the mating cycle in C. albicans also seems to occur in an atypical manner. Although reductional DNA divisions by a meiotic program have not been observed, tetraploid strains of C. albicans have been shown to return to the diploid state via a parasexual mechanism. During this process, tetraploid cells exposed to certain laboratory media were induced to lose chromosomes in an apparently random, but concerted, fashion, thereby forming cells with a diploid, or very close to diploid, DNA content [16]. The genetic locus responsible for determining C. albicans mating type (the *MTL* locus) segregated randomly in these experiments so that many of the progeny cells were **a** and α diploid cells that were themselves mating competent. Mating of diploid cells to form tetraploid cells, followed by random chromosome loss to generate diploid progeny cells, thereby constitutes a parasexual mating cycle in C. albicans.

In this study, we examined the genetic profile of strains formed by the parasexual mating process in C. albicans using SNP and comparative genome hybridization (CGH) techniques. We observed extensive shuffling of the parental configurations of chromosomes by the parasexual cycle, giving rise to many types of recombinant C. albicans progeny. Many of the progeny strains are not true (euploid) diploids; rather, they are aneuploid strains that are often trisomic for one or more chromosomes. In addition, we provide the first evidence that tetraploid strains experiencing chromosome instability and subsequent chromosome loss also undergo genetic recombination between homologous chromosomes.

We also report that genetic recombination in C. albicans tetraploids was dependent on the presence of Spo11p, a conserved protein that in other eukaryotes initiates meiotic recombination by the introduction of double-strand breaks (DSBs) into the DNA [17]. These results suggest that the parasexual pathway in C. albicans has evolved as an alternative pathway to meiosis for promoting a reduction in cell ploidy, and furthermore, that at least one gene that normally functions in meiotic recombination has been co-opted for use in the parasexual mating cycle.

## Results

### A Strain for Studying the Parasexual Mating Cycle in C. albicans

**Figure 1:**
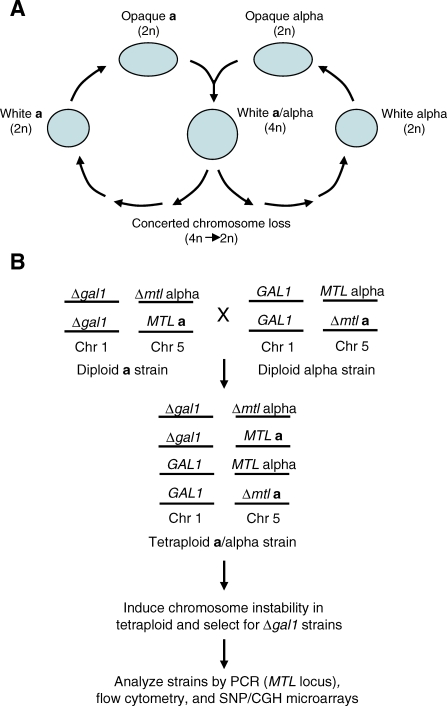
Analysis of the Parasexual Mating Cycle in C. albicans (A) Overview of the mating cycle in C. albicans. White *MTL**a*** and *MTL*α cells must switch to the opaque state to undergo mating and formation of a mononuclear tetraploid **a**/α cell. A reduction in ploidy back to the diploid (or near diploid) can occur by random chromosome loss. (B) A scheme for selection of diploid progeny strains from tetraploids. A tetraploid strain, RBY18, heterozygous for the *GAL1* gene on Chromosome (Chr) 1 and for all four *MTL* alleles on Chr 5 was constructed by mating *MTL**a*** and *MTL*α diploid strains, as shown. After induction of chromosome instability, strains that had undergone a reduction in ploidy were selected for by growth on 2-deoxygalactose (2-DOG) medium, as only strains that have lost the *GAL1* gene are able to grow on medium containing 2-DOG. Progeny strains were subsequently analyzed by PCR of the *MTL* locus and by flow cytometric analysis to confirm they were diploid strains. Strains were then analyzed by SNP and CGH microarrays to determine their genetic content.

The parasexual cycle of C. albicans, as currently envisaged, is shown in Figure 1A. Note that no meiotic program has been observed in C. albicans, despite the presence of many genes in the genome whose homologues function specifically in meiosis in other fungi [18]. However, C. albicans strains have been found to undergo a parasexual cycle; tetraploid strains become genetically unstable when incubated on certain laboratory media, losing chromosomes and generating diploid (and aneuploid) progeny strains that are themselves mating competent. The chromosome loss process is concerted, with loss of one or more chromosomes predisposing the cell to lose additional chromosomes, and the diploid state being the final product [16]. While tetraploids are stable when grown on YPD medium at different temperatures, two culture conditions were identified that induced genetic instability in C. albicans: (i) growth of tetraploid strains on S. cerevisiae “pre-sporulation” (pre-spo) medium at 37 °C, and (ii) growth of tetraploid strains on medium containing L-sorbose at 30 °C. The latter condition was previously shown to also induce chromosome loss in diploid C. albicans strains [19]. More specifically, diploid strains were unable to grow on L-sorbose medium unless they first underwent loss of one copy of Chromosome (Chr) 5, becoming monosomic for this chromosome. In contrast, diploid strains were relatively stable when grown on pre-spo medium, indicating that diploid and tetraploid strains exhibit very different selective pressures when cultured on this medium.

To monitor changes in ploidy in tetraploid strains of C. albicans, we exploited a genetically marked tetraploid strain, RBY18, containing markers on Chr 1 and 5. The strain was constructed by mating **a/**Δα and Δ**a**/α cell types, as shown in Figure 1B [16]. Strain RBY18 is heterozygous for the *GAL1* gene 1 on Chr 1, which is counterselectable. Strains carrying wild-type *GAL1* are unable to grow on medium containing 2-deoxygalactose (2-DOG) as the carbon source, while derivative strains that have lost both copies of the *GAL1* gene are able to grow on 2-DOG medium [20]. In most cases, it is expected that loss of *GAL1* function in RBY18 will occur by loss of both chromosomes carrying the *GAL1* allele, although *GAL1* function also can be lost by mutation or genetic recombination. The RBY18 tetraploid strain is also heterozygous for all four *MTL* alleles on Chr 5: WT**a**, WTα, Δ***a*** *1/**a**2*, and Δ*α1/α2*, which are easily distinguishable using whole cell PCR and oligonucleotides specific to each *MTL* allele [14].

### Selection of Diploid Progeny after Parasexual Chromosome Reduction

**Figure 2:**
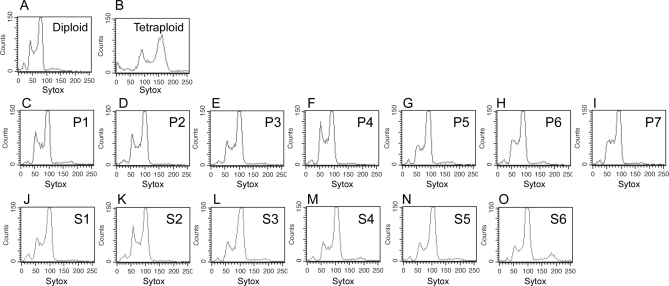
Analysis of Progeny Strains from the Parasexual Mating Cycle by Flow Cytometry Progeny strains derived from the tetraploid RBY18 were grown in liquid YPD medium, as described in Materials and Methods. Strains P1 to P7 (C–I) were derived from growth of RBY18 on pre-spo medium, while strains S1 to S6 (J–O) were derived from growth of RBY18 on sorbose medium. In both cases, progeny strains were found to be diploid, or near diploid, by flow cytometric analysis. For comparison, a parental diploid strain (A) and tetraploid strain (B) were also analyzed by flow cytometry. The *x*-axis of each graph (Sytox) represents a linear scale of nuclear fluorescence, and the *y*-axis (Counts) represents a linear scale of cell number.

To generate progeny strains that have undergone the parasexual mating cycle, the marked tetraploid strain RBY18 was induced to undergo chromosome loss on either pre-spo or sorbose medium and *gal1* ^−^ strains were selected by growth on 2-DOG medium. These 2-DOG resistant (DOG^R^) strains were subsequently analyzed by PCR to confirm that loss of *MTL* alleles on Chr 5 had accompanied loss of *GAL1* alleles on Chr 1, an indication that cells had undergone a reduction in overall cell ploidy (unpublished data). PCR of the *MTL* loci was also used to detect possible jackpot effects, where several *gal1* ^−^ progeny might have been derived from a single cell having undergone a chromosome loss event. Where possible, progeny cells with different combinations of *MTL* alleles were used for subsequent analysis. Selected progeny strains were grown in YPD medium at 30 °C and analyzed by flow cytometry to determine the overall ploidy of each strain, as shown in Figure 2. Flow cytometric analyses confirmed that each strain was diploid, or close to diploid, in DNA content, as judged by staining of the DNA with sytox green [9]. Seven strains (P1 to P7) were derived from RBY18 by growth on pre-spo medium, and six strains (S1 to S6) were derived from RBY18 by growth on sorbose medium (Figure 2). Subtle differences were observed in the flow cytometry DNA profiles between isolates, where distinct peaks were evident representing non-replicated (G1 phase) and replicated (G2 phase) DNA. In some strains the majority of the cells contained replicated DNA (e.g., S5 and S6, Figure 2, panels N and O), while others had an almost equal distribution of cells with unreplicated and replicated DNA (e.g., P4, panel F). However, there was no obvious correlation between DNA profiles analyzed by flow cytometry and cell growth rates.

**Figure 3:**
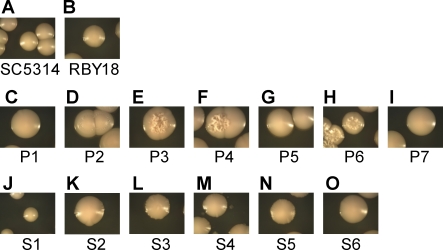
Morphology of Progeny Strains from the Parasexual Mating Cycle Progeny strains derived from the tetraploid RBY18 strain by growth on pre-spo medium (P1 to P7) or sorbose medium (S1 to S6) were analyzed on YPD medium. Strains were grown at 30 °C for 7 d and photographed. Many strains exhibited a mutant morphology, including increased surface wrinkling of the colonies indicative of increased hyphal cell formation. A control diploid strain (SC5314) and tetraploid strain (RBY18) are included for comparison.

To further characterize the strains generated by parasexual chromosome reduction, progeny were plated for single colonies on rich (YPD) medium to examine colony growth. After incubation at 30 °C for 7 d, colonies were compared for overall size and morphology (Figure 3). A wide range of phenotypes was observed, including smaller colony sizes relative to diploid and tetraploid parental strains and altered colony morphologies. Some of the isolates produced hyper-filamentous morphologies, as evidenced by increased surface wrinkling of the colonies (e.g., progeny strains P3, P4, and P6; Figure 3, panels E, F, and H). Normally, C. albicans cells grow as budding yeast, pseudohyphal, or true hyphal cells. Examination of cells from the wrinkled colonies by microscopy confirmed that these colonies contained many filamentous (pseudohyphal and true hyphal) cells, while the unwrinkled colonies (including control strains) contained very few filamentous cells (unpublished data). Some progeny strains also exhibited reduced filamentation on medium that normally induces hyphae formation (Spider medium and serum-containing medium, KA and RJB, unpublished data).

Thus, the parasexual cycle of C. albicans can generate variant strains with diverse colony morphologies. Changes in the ability to undergo the yeast-hyphal transition have been closely linked with the pathogenic potential of C. albicans strains [21–24]. It is therefore likely that many of these variant strains will exhibit reduced virulence in models of candidiasis; but it is also possible that some of these isolates could have increased fitness under particular selective conditions, leading to improved colonization of defined in vivo niches in the host.

### Genomic Profiling of Progeny Cells from the Parasexual Cycle

SNP and CGH microarrays are powerful approaches for examining genetic recombination and genome structure in C. albicans [25–28]. SNP arrays were designed to exploit the sequence diversity between chromosome homologues in the diploid C. albicans genome. The genome-wide SNP arrays used here included 152 SNPs, distributed across all eight chromosomes of C. albicans. As each SNP is specific for one of the parental chromosome homologues, each homologue can be distinguished in progeny from the parasexual mating cycle. In addition, loss of heterozygosity (LOH) at SNPs on otherwise heterozygous chromosomes can be used as a marker for genetic recombination. Quantitative SNP analysis can also be used to determine the relative copy number of each homologue in a sample (see Materials and Methods).

CGH analysis provides a complementary approach to SNP arrays for the determination of the copy number of each gene on each chromosome in the sample. Labeled genomic DNA from experimental samples (Cy3 labeled) and labeled DNA from a reference diploid SC5314 strain (Cy5 labeled) were hybridized to whole genome arrays containing >6,000 C. albicans ORFs [27,28]. CGH data provides information on the copy number of every chromosome, as well as indicating large-scale aneuploidies. In this study, we used both CGH and SNP approaches to obtain a detailed picture of the products of the C. albicans parasexual cycle following concerted chromosome loss.

SNP and CGH arrays were first used to analyze RBY18 and the diploid parental strains that had been used to construct this tetraploid strain. SNP analysis confirmed that *MTL**a*** and *MTLα* parental diploid strains were heterozygous for most of the SNPs on the array, although in the parental *MTLα* strain Chr 2 was homozygous for all markers (Table S4). CGH array data confirmed that the parental strains were euploid diploids and RBY18 was a euploid tetraploid, as they contained two and four copies of each of the eight C. albicans chromosomes, respectively.

**Figure 4:**
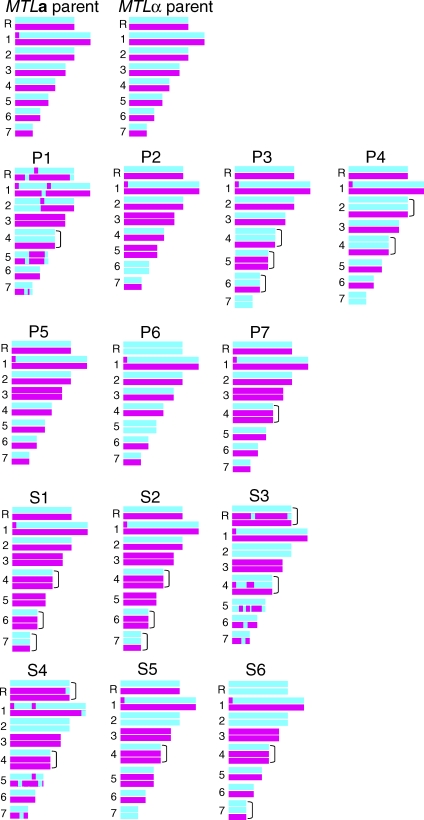
Schematic Summary of Genomic Profiles of Progeny Strains Derived from Tetraploid Strains via the Parasexual Cycle Progeny strains were analyzed by SNP and CGH whole-genome microarrays to determine the copy number of each chromosome and the configuration of chromosome homologues. Chromosome homologues are indicated by blue and pink bars to represent “maternal” and “paternal” homologues, respectively. Genetic recombination events are indicated by loss of heterogeneity between chromosome homologues. In cases where the chromosome is trisomic, this is indicated by a bracket to the right of the trisomic chromosome. P1 to P7 progeny strains were derived from growth of the tetraploid RBY18 strain on pre-spo medium, while S1 to S6 strains were derived from tetraploid growth on sorbose medium. Detailed SNP and CGH array data is provided in Table S4 and Figure S4.

We then analyzed 13 progeny strains produced by concerted chromosome loss from RBY18 using SNP and CGH arrays (see Figures 4 and S4, and Tables S1 and S4). Only three of the 13 strains were true diploids (P2, P5, and P6). The majority (10/13) of the progeny strains contained at least one extra chromosome: four of the seven strains derived from growth of the tetraploid on pre-spo medium were trisomic for one to three chromosomes and all six strains derived from growth on sorbose were also trisomic for up to three of the eight C. albicans chromosomes (Figure 4). Thus, concerted chromosome loss was often incomplete and did not immediately result in true diploid strains.

Curiously, there was a strong bias towards trisomy of Chr 4 in the progeny strains; all strains carrying at least one trisomic chromosome (four pre-spo-selected strains and all sorbose-selected strains) were trisomic for Chr 4. Trisomies of Chr R, 2, 5, 6, or 7 were also detected in at least one of the progeny. As expected, Chr 1 was always present in the disomic parental configuration (one copy of each homologue) because selection of DOG^R^ progeny requires that the strains lose both Chr 1 homologues from the *MTL*α mating parent (Figure 1B).

### Genetic Recombination in the Parasexual Cycle of C. albicans

The most striking feature of the progeny genetic profiles was that three strains contained a number of short LOH tracts (six or seven LOH tracts were observed in each strain), evidence of multiple recombination events between homologous chromosomes. Isolates P1, S3, and S4 exhibited recombination events that included LOH at SNPs on multiple chromosomes (including Chr R, 1, 2, 4, 5, 6, and 7, see Figure 4). While selection on 2-DOG required inheritance of the *gal1*Δ alleles on Chr 1, the LOH events detected here are independent of the *GAL1* locus. Moreover, these events did not involve homozygosis of all of Chr 5, which might be expected to occur in response to sorbose selection. Instead, the recombination events we observed appear to be selection independent. Overall, the appearance of multiple gene conversion tracts within several strains, and the general absence of gene conversion tracts in other strains, suggests that some cells become generally competent for recombination at more than one locus, while other strains do not undergo such recombination events at all.

In at least one example (Chr 2 in strain P1) one complete chromosome arm (Chr 2L) became homozygous (Figure 4). This recombination event may have arisen in one of two ways: (i) A cross-over between chromosomes led to reciprocal recombination between homologues, as commonly occurs during meiosis in other fungi. In this case, the partner DNA involved in the reciprocal exchange was lost during the process of concerted chromosome loss. (ii) A break-induced replication event occurred. In this case, a DSB in one chromosome was repaired by DNA replication that copied the template strand from the break near the centromere all the way to the telomere in the homologous chromosome. Break-induced replication is a non-reciprocal recombination event and in S. cerevisiae is often restricted to repair of DNA DSBs where only one end of the break shares homology with the template [29].

Potential hotspots for recombination were identified in the three strains that had undergone inter-homologue recombination. For example, SNPs HST3 and 2340/2493 on Chr 5 underwent LOH in P1, S3, and S4 recombinant strains. Additional experiments are necessary to fully document hotspots for recombination. However, our results indicate that recombination events are not uniform across the C. albicans genome during the parasexual cycle.

### Does the C. albicans Genome Harbor Recessive Lethal Alleles?

Natural isolates of C. albicans are diploid, and it has been proposed that haploid forms cannot exist because of the presence of recessive lethal alleles in the genome. Evidence supporting this idea came from classical mitotic recombination studies [30,31]; however, no systematic investigation of possible recessive lethal alleles in the C. albicans genome has been reported. Using the present dataset, we can rule out the presence of recessive lethal alleles on some chromosomes. For example, it was already known that Chr 5 does not harbor recessive lethal alleles: loss of either homologue can be induced in diploid cells by growth on sorbose medium [19]. The SNP data presented here supports this finding, as both AA and BB configurations of Chr 5 homologues were observed in the progeny strains P2 and P6, respectively (this nomenclature assigns the parental configuration of chromosome homologues as AB). Similarly, several other chromosomes did not carry recessive lethal alleles, as their homologues could be lost during the parasexual cycle. Chr R, 2, 3, 5, 6, and 7 were all found to be homozygous in at least one independent isolate. However, only one homozygous configuration was observed for each chromosome (either AA or BB), leaving open the possibility that the other chromosome homologue carries recessive lethal alleles. We will revisit the issue of recessive lethal alleles below.

### The Role of Meiosis Genes in C. albicans

The C. albicans parasexual cycle provides an alternative mechanism to meiosis for a reduction in cell ploidy. Although no experimental evidence for a meiotic pathway in C. albicans currently exists, the genome contains homologues of many genes that function specifically in meiosis in the related yeast S. cerevisiae [18]*.* Some of the meiosis genes from C. albicans even complement for meiotic function in S. cerevisiae, demonstrating they encode a conserved protein activity [32]. It seems likely that either (i) C. albicans has a cryptic meiotic program still to be discovered, or (ii) meiotic genes have been adapted to other processes in C. albicans, perhaps some in the parasexual pathway.

To address the latter possibility, we investigated the potential role of the Spo11 protein in genetic recombination during the parasexual cycle. In fungi such as S. cerevisiae and S. pombe and in higher eukaryotes, Spo11p makes meiosis-specific DSBs in DNA via a topoisomerase-like mechanism of DNA cleavage [33,34]. *C. albicans ORF19.11071* on Chr 2 encodes a potential homolog of *S. cerevisiae SPO11* (http://www.candidagenome.org). An alignment of this ORF with *SPO11* genes from diverse species including S. pombe, S. cerevisiae, Kluyveromyces lactis, and *Drosophila* reveals that several of the critical conserved residues identified for DNA strand cleavage are present in the C. albicans sequence (Figure S1). In particular, the conserved active site tyrosine residue, required for breakage of the DNA and formation of a phosphotyrosine bond, is present in the C. albicans protein. Similarly, Glu-233 and Asp-288 residues that are required in S. cerevisiae Spo11p for meiotic recombination [35] are conserved in the C. albicans protein. *ORF19.11071* is a homologue of the Spo11 family and will therefore be referred to as C. albicans Spo11p in the rest of this study. Attempts to complement S. cerevisiae Spo11 function with C. albicans Spo11p, as measured by rates of meiotic recombination in return-to-growth experiments, were unsuccessful (Table S2). This result is perhaps not surprising as *SPO11* sequences from diverged species are poorly conserved outside of the core catalytic residues [36] (Figure S1). It is also worth noting that meiotic proteins in general are faster evolving than most cellular proteins [37,38], an issue that is taken up again in the Discussion.

**Figure 5:**
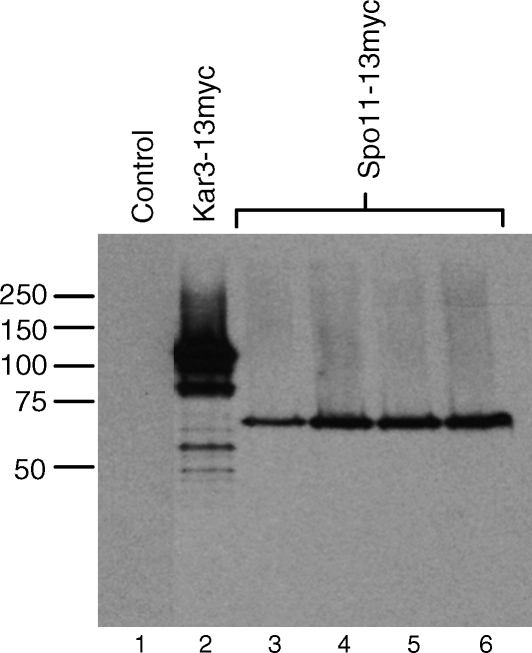
Expression of C. albicans Spo11 Protein in Mitotic Cells The expression of a Spo11–13 × myc-tagged protein was analyzed by western blotting. Lane 1 shows a control strain lacking the Spo11-13myc fusion construct, while lane 2 shows expression of a Kar3-13myc protein for comparison. Lanes 3–6 show extracts from four independently transformed diploid strains with the Spo11-13myc construct (see Materials and Methods).

To investigate whether C. albicans Spo11p is expressed in mitotically dividing cells, a Spo11-13myc fusion protein was constructed in diploid C. albicans strains. Western blots show that the Spo11-13myc protein was detectable in mitotic extracts of diploid cells grown in YPD medium, although the level of expression was relatively low (see comparison of protein levels with that of the mitotic spindle protein Kar3-13myc) (Figure 5). Thus, in C. albicans, the Spo11 protein is expressed in mitotically dividing cells.

### Genetic Recombination in the C. albicans Parasexual Mating Cycle Is Dependent on Spo11 Function

**Figure 6:**
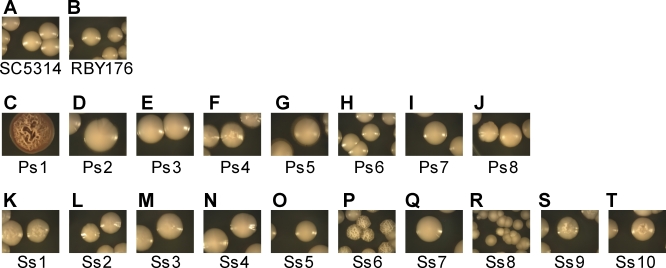
Morphology of Diploid Progeny Strains Derived from the Δ*spo11* Tetraploid Strain Progeny strains were grown on YPD medium at 30 °C for 7 d and colonies photographed. Progeny strains Ps1 to Ps8 were derived from growth of the Δ*spo11* tetraploid strain on pre-spo medium, while strains Ss1 to Ss10 were derived from growth on sorbose medium. A control diploid strain (SC5314) and tetraploid strain (RBY176) are shown for comparison.

The observation that C. albicans Spo11p is expressed during mitotic growth is consistent with it having a function outside of meiosis. To examine if C. albicans Spo11p is required for genetic recombination in the parasexual mating cycle, we deleted all four copies of the *SPO11* gene in genetically marked tetraploid strains (RBY176/RBY177) that were heterozygous for *GAL1* on Chr 1. The strains were induced to undergo concerted chromosome loss on pre-spo or sorbose medium and were then exposed to 2-DOG to select for strains that had lost both copies of *GAL1*. Eighteen DOG^R^ colonies were selected from tetraploid growth on pre-spo (eight colonies) or sorbose (ten colonies) and subsequently analyzed by flow cytometry to determine if they were diploid, or near diploid, strains (Figure S2). Indeed, we detected diploid Δ*spo11* progeny strains, indicating that Spo11p is not necessary for the process of concerted chromosome loss in tetraploid C. albicans strains. We next analyzed the colony morphologies of the Δ*spo11* diploid progeny. As was seen with progeny from wild-type tetraploids (Figure 4), many of the Δ*spo11* progeny strains exhibited altered colony morphologies on YPD medium (Figure 6).

**Figure 7:**
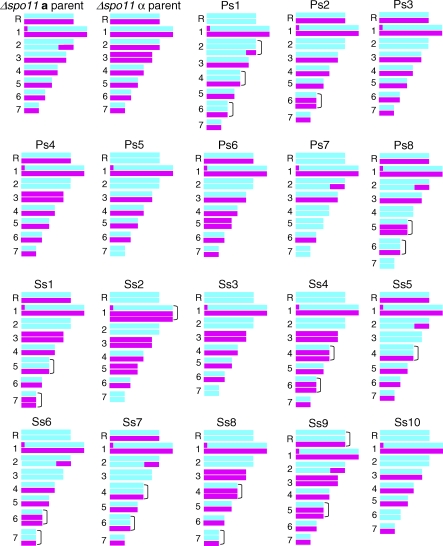
Schematic Summary of Genomic Profiles of Progeny Diploid Strains Derived from the Δ*spo11* Tetraploid Strain via the Parasexual Cycle Progeny strains were analyzed by SNP and CGH microarrays to determine the genetic content of each strain. As described in the legend to Figure 4, chromosome homologues are indicated by blue and pink bars to represent “maternal” and “paternal” homologues, respectively. In cases where a chromosome is trisomic, this is indicated by a bracket to the right of the chromosome. Ps1 to Ps8 progeny strains were derived from growth of the Δ*spo11* tetraploid (RBY176 or RBY177) on pre-spo medium, while Ss1 to Ss10 strains were derived from Δ*spo11* tetraploid growth on sorbose medium. Detailed SNP and CGH array data are provided in Tables S5 and S6 and Figure S4.

Genomic profiles of the Δ*spo11* diploid progeny (along with the parental diploid and tetraploid strains) were generated using SNP and CGH microarrays (see Figure 7, as well as Tables S5 and S6, and Figures S1 and S4). One of the diploid parents (RBY79, *MTL*α parent) was initially homozygous for Chr 2, and the other parent (RBY77, *MTL* **a** parent) carried a long tract of LOH on Chr 2 (Figure 7). This is reflected in the patterns of Chr 2 inheritance in the diploid progeny which either received only one type of Chr 2 homologue (Ps2, Ps3, Ps4, Ps5, Ps6, Ss1, Ss2, Ss3, Ss4, Ss8, and Ss10) or received two homologues that only differ near the Chr 2R telomere (Ps1, Ps7, Ps8, Ss5, Ss6, Ss7, and Ss9). Similarly, one of the *gal1*Δ Chr 1 homologues in the parental *MTL**a*** strain had undergone LOH of a single SNP near the telomere of Chr 1L and this LOH tract was retained in all of the progeny.

As in the wild-type (*SPO11^+^*) progeny that were close to diploid, a majority (11/18) of the strains carried at least one and up to three trisomies, and Chr 4 was often one of the trisomic chromosomes (5/11 strains). Other chromosomes that became trisomic were Chr R, Chr 1, Chr 2, Chr 5, Chr 6, and Chr 7. The only chromosome that did not become trisomic in these strains or in the wild-type diploid progeny strains was Chr 3. Concerted chromosome loss did result in homozygosis of Chr R in nine strains (and trisomy in one strain) with the same homologue always being retained (the blue-colored “A” homologue in Figure 7). Interestingly, while no trisomies of Chr 3 were found, Chr 3 underwent LOH in ten strains, with seven of them retaining homologue B (colored pink) and three retaining the A homologue (Figure 7).

The most striking feature of the Δ*spo11* progeny strains was that they did not undergo any detectable genetic recombination events. No single LOH events (gene conversion events) or chromosome crossing over events (long-range LOH) events were observed (although we note that if reciprocal recombination events occurred, in which both recombinant chromosomes were retained, these would not be detected by SNP analysis). In contrast, progeny derived from *SPO11^+^* strains exhibited multiple recombination events in three out of 13 strains (Figure 4), a difference that is statistically significant (*p* < 0.05). Taken together, the SNP and CGH experiments indicate that genetic recombination takes place in wild-type cells during the parasexual mating cycle, generating recombinant C. albicans strains. These recombination events are dependent on Spo11p, a conserved protein that normally acts specifically in meiosis in a wide range of eukaryotes. We suggest that Spo11p function has been adapted in C. albicans for mediating genetic recombination in the alternative parasexual mating cycle.

### Analysis of the Pattern of Concerted Chromosome Loss during the Parasexual Cycle

The Spo11 experiment more than doubled the data on chromosome loss from C. albicans tetraploids, and we used this expanded dataset to re-evaluate patterns of chromosome loss during the parasexual cycle. We pooled genomic profiling data for all 31 progeny strains derived from tetraploids by concerted chromosome loss (13 from wild-type *SPO11^+^* tetraploids and 18 from Δ*spo11* tetraploids) (Table 1). We determined, for each chromosome in each strain, whether they existed as the parental configuration of homologues (AB), a homozygous configuration (AA or BB), or a trisomic configuration (AAB or ABB). We excluded Chr 1 from this analysis, as selection for the loss of the *GAL1* gene required the AB configuration be retained for Chr 1 in all isolates (see Figure 1B).

**Table 1:**
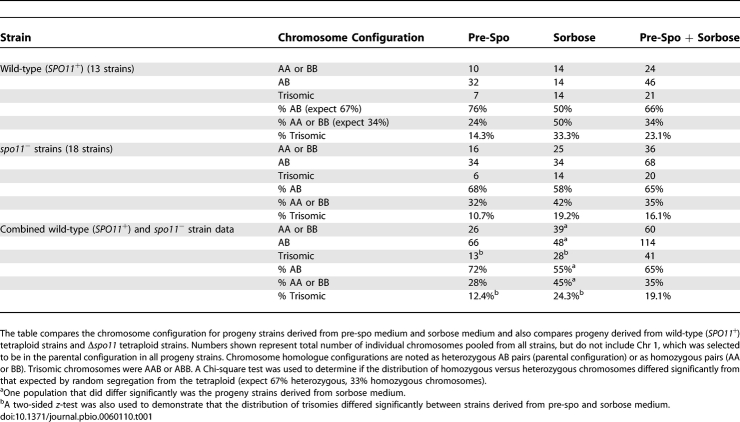
Summary of Genotypes in Progeny Strains Derived from the Parasexual Mating Cycle

While the number of strains analyzed in this study is relatively small, several trends are apparent. First, if two of the four copies of each chromosome in tetraploid strains were lost with equal probability, it would be expected that 67% of chromosomes would consist of AB homologues, while 33% of chromosomes would exhibit either AA or BB configurations. Isolates selected from pre-spo medium contained a chromosomal distribution very close to this, with 72% of disomic chromosomes being AB homologues, and 28% of chromosomes being AA or BB homologues. In contrast, isolates derived from sorbose medium were biased towards a homozygous chromosome configuration (45% were AA or BB with only 55% exhibiting the AB configuration). Sorbose-selected strains were also more likely to contain trisomic chromosomes than were strains selected on pre-spo medium. Trisomic chromosomes were present for 24.3% of chromosomes selected on sorbose medium, while only 12.4% of chromosomes were trisomic in isolates selected on pre-spo medium. Both of these differences between pre-spo and sorbose media were significant (*p* < 0.05) and provide evidence that strains undergoing chromosome loss on these media either experience different patterns of chromosome loss or different selective pressures.

Previous studies have also observed differences between pre-spo and sorbose medium in that diploid C. albicans strains were stable on pre-spo medium but exhibited chromosome instability (particularly that of Chr 5 but also of other chromosomes) on sorbose medium [16,19,39]. One possibility for the higher fraction of homozygous AA/BB chromosomes in tetraploids exposed to sorbose medium is that these conditions generate monosomic chromosomes that then undergo re-duplication to form homozygous disomic chromosomes. At least for Chr 5, this possibility was ruled out by PCR typing of *MTL* alleles on this chromosome, as all four *MTL* alleles in the tetraploid are distinct (*MTL**a***, *MTLα*, *MTLΔ**a***, *MTLΔα*; Figure 1B). PCR analysis revealed that strains that were homozygous for Chr 5 by SNP analysis always contained two distinct *MTL* alleles, indicating that monosomy and reduplication had not occurred (unpublished data). These experiments demonstrate that, at least for tetraploid strains, the formation of viable progeny on sorbose medium does not require monosomy of Chr 5 at any stage.

Trisomy was more common in strains derived from sorbose medium than pre-spo medium and could be due either to chromosome loss of one homologue from tetraploids or to re-duplication of one chromosome homologue in a disomic strain. Curiously, at least in a subset of cases, trisomy was a result of reduplication of one chromosome homologue in sorbose-derived strains. For example, three strains, S5, Ss1, and Ss9, were shown to be trisomic for Chr 5 by SNP analysis and yet each strain contained only two types of *MTL* allele by PCR genotyping. This indicates that trisomy of Chr 5 arose by re-duplication of one homologue of Chr 5 in a disomic strain. In addition, one isolate (strain Ss2 derived from sorbose medium) was trisomic for Chr 1, but was clearly *gal1*− by PCR (lacked the *GAL1* ORF) and was also 2-DOG resistant. Thus, where trisomies can be distinguished in sorbose-derived strains, they were due to re-duplication of chromosome homologues for Chr 1 and Chr 5. In contrast, strain Ps8 derived from pre-spo medium was trisomic for Chr 5 and also tri-allelic at the *MTL* locus, indicating that trisomy occurred via loss of one homologue of Chr 5 and not chromosome re-duplication. Overall, our results suggest that growth of tetraploids on sorbose medium may apply more selective pressure to the cells than growth on pre-spo medium, causing them to produce progeny with increased trisomies by chromosome re-duplication and more bias in the distribution of whole chromosome LOH events.

A further conclusion from our analysis is that every chromosome (excluding Chr 1 because of the selection for *gal1*Δ/Δ) can exist in a homozygous form. Because of the limited number of strains analyzed, we only detected one homozygous configuration (either the AA or BB configuration) for most chromosomes, however both Chr 3 and Chr 5 were found in both the AA and BB configurations. This implies that Chr 3, like Chr 5, does not contain recessive lethal alleles on either chromosome homologue. One possible caveat to this conclusion is that undetected recombination events may have repaired recessive lethal alleles on these chromosome homologues. However, this seems unlikely given that the *Δspo11* progeny produced a significant number of strains that were homozygous for both homologues of Chr 3; seven progeny were homozygous for the B homologue and three were homozygous for the A homologue.

We detected trisomies for all chromosomes except Chr 3 in at least one progeny strain. This suggests that one or more genes on Chr 3 may not be well tolerated at higher than euploid copy number under these conditions. Recent studies in S. cerevisiae have shown that increased copy numbers of certain chromosomes can be lethal, as haploid cells disomic for Chromosome VI were inviable [40]. However, at least in the majority of cases, C. albicans strains trisomic for one or more chromosomes were viable and produced apparently stable karyotypes.

Finally, a comparison of the growth rates of the progeny strains from the parasexual cycle was revealing. While most of the euploid progeny grew at rates very similar to that of a control diploid strain, aneuploid strains grew at increasingly slower rates as the number of trisomic chromosomes increased (Figure S3). Thus, euploid progeny grew on average 7.4% slower than a control SC5314 strain, while strains containing one trisomic chromosome grew 9.5% slower, strains containing two trisomies 16.3% slower, and strains with three trisomies 23.4% slower. Thus, as the number of additional chromosomes increased, so, in general, did the doubling time of the cell. In S. cerevisiae a similar observation has been made, where aneuploid chromosomes (disomies in haploids or trisomies in diploids) were found to cause a proliferative disadvantage, and this disadvantage generally increased as the number of extra chromosomes increased [40,41]. Aneuploidy therefore appears to confer a proliferative disadvantage in multiple yeast species.

## Discussion

Most sexually reproducing organisms use meiosis to reduce the chromosome number of the cell and to generate genetic diversity through recombination, typically for the formation of recombinant haploid progeny from diploid precursors. In C. albicans, a meiotic program has not been identified, despite an intact mating apparatus and the presence of many genes in the genome that function specifically in meiosis in related fungal species [18]. However, an alternative pathway has been described that through chromosome loss can complete a parasexual cycle. In contrast to the precision of the meiotic process, the parasexual pathway utilizes random, yet concerted, chromosome loss for the formation of diploid progeny from tetraploid cells [16]. In this paper, we used both SNP and CGH microarray analyses to reveal that the parasexual pathway generates highly divergent strains by three distinct mechanisms: (i) shuffling of whole chromosomes, leading to new combinations of homologues; (ii) formation of aneuploid strains, usually trisomic for one or more intact chromosomes; and (iii) accumulation of multiple recombination events between homologous chromosomes, in a process that is dependent on the conserved meiosis protein, Spo11p.

The parasexual process of concerted chromosome loss in C. albicans was previously suspected of yielding new combinations of homologues by random segregation of parental chromosomes [16,42]. Our work now confirms this idea. Strains from the parasexual cycle also often contained aneuploid chromosomes, as would be expected if the chromosome reduction process was not completed or if strains obtained a selective advantage from the presence of a particular aneuploidy (as suggested by the prevalence of Chr 4 trisomies in this study). Prior to the discovery of mating in C. albicans, classical experiments demonstrated that tetraploids could be formed by fusion of spheroplasted diploid cells [43,44], and that chromosome instability could be induced in these tetraploid cells by artificial means (e.g., heat shock, drug selection) [45,46]. The products of chromosome loss were also cells with a diploid, or close to diploid, DNA content, indicating that random segregation of chromosomes can occur in tetraploids generated either by mating or by fusion of spheroplasted cells.

Much less expected was our discovery that some strains undergoing the parasexual cycle underwent genetic recombination between homologous chromosomes. Even more surprising, these strains underwent recombination events at multiple different chromosomal loci. The observation that recombination was extensive in some strains but absent in others, may indicate that strains can exist in two alternative states; those that are primed to undergo genetic recombination during the parasexual cycle and those that are not. We note that it is unlikely that these recombinant strains were formed by a subset of tetraploids undergoing meiosis, as all three recombinants were aneuploid, being trisomic for at least one chromosome. In contrast, meiosis would be expected to produce primarily true diploid (euploid) strains without chromosomal aneuploidies. Genetic recombination is integral to meiosis in most fungi, where accurate segregation of chromosomes at the first meiotic division requires recombination between homologous chromosomes (for recent reviews see [17,47,48]). Meiotic recombination is initiated by the formation of DNA DSBs catalyzed by Spo11p, generating covalent protein-DNA intermediates that are subsequently processed by enzymes including homologs of bacterial RecA [49,50]. Meiotic recombination can lead either to the reciprocal exchange of DNA flanking the DSB (crossover events) or to events in which no exchange of the flanking DNA takes place (gene conversion or non-crossover events). However, at least in S. cerevisiae, Spo11 has not been observed to influence rates of mitotic recombination (C. Giroux, personal communication).

Recombination in strains undergoing the parasexual pathway in C. albicans was less frequent than that expected from a classical meiotic pathway. Significantly, however, deletion of C. albicans Spo11 function eliminated all recombination during the parasexual mating cycle (Figure 7 and Tables S5 and S6). This result suggests that C. albicans Spo11p is integral to the generation of genetic diversity during the parasexual cycle, and thereby enhances the degree of variability in the strains produced. In addition, in the absence of evidence for a functional meiosis in C. albicans, our findings suggest a role for the conservation of one meiotic gene in this organism. We propose that the Spo11 protein, which functions specifically in meiosis in other organisms, has been re-programmed in C. albicans to function during the parasexual pathway. Future experiments will determine if other meiosis-specific proteins function in the alternative parasexual process in C. albicans. We cannot rule out, however, the possibility that meiotic proteins have been retained to function in a cryptic meiotic pathway that remains to be discovered.

In S. cerevisiae, Spo11 functions together with a number of accessory proteins to introduce meiotic DSBs, including Ski8, Mer2, Mei4, Rec102, Rec104, and Rec114 [17]. However, with the exception of Ski8, homologues of these accessory factors are not recognizable in the C. albicans genome [18]. This may be due, at least in part, to the fact that many of the proteins involved in meiotic recombination are faster evolving than most other cellular proteins [37,38]. The limited conservation of meiotic factors may also account for the observation that C. albicans Spo11p did not complement an *S. cerevisiae spo11* mutant for meiotic recombination. In fact, cross-complementation of Spo11p function between any two species has yet to be successfully demonstrated (S. Keeney, personal communication). Even in S. pombe, where a number of genes involved in meiotic DSB formation have been identified, most of these genes share either very limited or no sequence homology with genes in S. cerevisiae or any other organism. Thus, many of the proteins involved in DSB formation appear to have significantly diverged from one another. In addition, it appears that different biochemical functions are utilized in different organisms to initiate the formation of DSBs [17]. Clearly, it will be of significant interest to identify co-factors that act with Spo11 to mediate recombination during the parasexual cycle of C. albicans.

### Phenotypic Analysis of Parasexual Progeny Strains

The products of the C. albicans parasexual cycle were diploid and aneuploid progeny that exhibited altered colony morphology phenotypes. In particular, many strains had an increased tendency to form hyphal filaments on solid medium, evident either by increased surface wrinkling of the colony or by increased peripheral filamentation at the edge of the colony. Since the yeast-hyphal transition is closely associated with virulence of C. albicans strains, it is likely that many of these progeny strains will show altered virulence in animal models of candidiasis. Several of the progeny strains exhibited growth defects relative to control diploid strains, to control tetraploid strains, and to other diploid progeny. In some cases this was likely due to chromosomal aneuploidies, as many of these strains carried extra copies of up to three of the eight chromosomes of C. albicans. Indeed, being trisomic for two or three chromosomes increased cell doubling times by 16% and 23%, respectively, over a diploid control strain. Recent studies in S. cerevisiae have found that aneuploidy due to the presence of one or more additional chromosomes resulted in compromised growth rates [40]. Aneuploidy of large chromosomes or of multiple chromosomes correlated with the most significant cell cycle delays in S. cerevisiae [40]. We observed the same phenomenon in C. albicans strains, that the more aneuploid chromosomes a strain carries, the greater the proliferative disadavantage.

Compromised growth was also observed in a subset of euploid progeny from the parasexual mating cycle. In general, it appeared that LOH on 1–2 chromosomes did not typically compromise growth rates, but that LOH across multiple chromosomes did (e.g., strains Ps7 and Ss10). In these cases, it is likely that LOH at multiple genes led to the reduced fitness of these strains. Consistent with this idea, most clinical isolates, including the SC5314 strain whose genome was sequenced, show extensive heterozygosity. Previous studies have shown that allelic differences between C. albicans genes from different chromosome homologues can result in altered protein expression and altered protein function [51–54]. In addition, a recent study analyzed Chr 5 heterozygosity in multiple clinical isolates and found that LOH at multiple genes along Chr 5 reduced the virulence of strains in a model of systemic candidiasis [55]. Our work is also consistent with the idea that heterozygosity of multiple chromosomes provides C. albicans strains with a fitness advantage, at least for growth on laboratory media. Taken together, these results indicate that being heterozygous for genes on multiple chromosomes can improve both the fitness of mitotically dividing cells in vitro and the virulence of strains in vivo. However, the parasexual cycle (including the recombination events described in this paper) generates a great deal of genetic diversity, and it seems likely that conditions exist where strains that show reduced fitness in the laboratory have a selective advantage elsewhere.

### Sexual Versus Asexual Reproduction

Recent work has revealed that C. albicans, like other prevalent human fungal pathogens such as Cryptococcus neoformans and Aspergillus fumigatus, has access to a sexual mating program, but that under most conditions it propagates primarily in an asexual manner [56–58]. Recent studies in the model yeast S. cerevisiae found that both asexual and sexual modes of reproduction can be advantageous under the right experimental conditions. Under constant environmental conditions, the asexual mode of propagation was favored, but under stressful conditions the sexual strain had the competitive advantage [59,60]. In the case of C. albicans strains, population genetics on clinical isolates first suggested that the predominant mode of reproduction was clonal, with only limited evidence for genetic recombination between strains [61–64]. A recent study, however, found evidence for a high frequency of recombination events amongst clinical isolates, consistent with C. albicans strains undergoing sexual or parasexual recombination in their natural environment [13]. These studies are also consistent with the results presented here: the parasexual mating cycle can generate variant genotypes, including a subset of strains that have undergone extensive genetic recombination between chromosomes.

For C. albicans, the parasexual mechanism may provide two significant benefits over a conventional sexual pathway. First, the parasexual mechanism is imprecise, generating many aneuploid strains as well as euploid progeny strains. Common aneuploidies included diploid strains harboring trisomic chromosomes, and this karyotypic variation led to greater genetic and phenotypic diversity in the progeny population. Consistent with this observation, changes in chromosome copy number have previously been linked to phenotypic changes in C. albicans, including increased resistance to antifungal azoles [28,65]. Thus, karyotypic variation appears to be an important mechanism utilized by C. albicans to regulate physiologically important genes [66]. In S. cerevisiae, aneuploid strains are similarly at a competitive disadvantage with euploid strains, unless there is a strong selective pressure that favors growth of the aneuploid form [40,67,68]. A second potential benefit of the parasexual cycle is that it bypasses the process of sporulation common to the sexual cycle of most ascomycetes. Ascospores are thought to be highly antigenic, and, given that C. albicans strains normally exist as commensals within warm-blooded hosts, the absence of spore formation may facilitate the generation of genetic diversity without compromising cell survival [56].

In summary, the parasexual cycle in C. albicans provides an alternative to a sexual reproductive cycle. Concerted chromosome loss reduces the ploidy of the cell from tetraploid to approximately diploid, generating recombinant progeny strains with variant phenotypes. Genetic recombination between homologous chromosomes, dependent on the Spo11 protein, takes place during these reductive mitotic divisions, further contributing to genetic diversity. We propose that at least some of the meiotic recombination machinery has been re-programmed to function in parasexual recombination in C. albicans. Finally, we note that as C. albicans thrives only in warm-blooded animals, the parasexual cycle provides a number of potential advantages over a conventional sexual cycle.

## Materials and Methods

### Strains and media.

Standard laboratory media were prepared as previously described [69]. Construction of the genetically marked tetraploid strain, RBY18, was previously described [16]. A tetraploid Δ*spo11* strain was constructed by first deleting the *SPO11* gene in the diploid strains RBY16 and CHY477 [16]. Both copies of *SPO11* were sequentially disrupted using a modified Ura blaster method [70,71]. A *SPO11* gene disruption construct was made by PCR amplifying the *HisG-URA3-HisG* cassette using oligonucleotides SPO11 KO-5′ and SPO11 KO-3′ from plasmid pDDB57 (see Figure S5) [71]. Heterozygous strains were then constructed by replacing *SPO11* coding sequences with the *URA3* selectable marker flanked by *HisG* repeats. Ura+ strains that were deleted for one copy of *SPO11* were grown on nonselective medium and subsequently plated on SCD medium containing 5-fluoroorotic acid (5-FOA) and uridine medium to select for loss of the *URA3* gene [70]. The *HisG-URA3-HisG* cassette was then used to delete the second copy of the *SPO11* gene. The construction of *Δspo11* mutants was confirmed using PCR to check both 5′ and 3′ junctions following integration of the Ura blaster cassette and also to confirm the loss of the *SPO11* ORF following the second round of transformation. Deletion of *SPO11* in the diploid strains RBY16 and CHY477 generated strains RBY77 and RBY79, respectively. The diploid strains were mated as previously described [16] to form the tetraploid Δ*spo11* strains RBY176 and RBY177.

To follow expression of the Spo11 protein the gene sequence was fused to that encoding a 13 × myc epitope tag. The Spo11 gene and promoter were first amplified by PCR using oligonucleotides Spo11(myc) for, 5′-cccaatatgaagcactaaactc-3′ and Spo11(myc) rev, 5′-ggcgcgcccggggatccgtttcgtatagctagccgttcc-3′. The amplified sequence was then digested with HindIII and SmaI enzymes and ligated into a pMYC-HIS1 vector. The resulting plasmid contains the *SPO11* gene sequence fused to 13 copies of the myc epitope. The plasmid was then linearized by digestion with BstBI and used to transform strain RBY1118 (a diploid **a**-type mating strain) to generate CAY126. RBY1118 itself was derived from **a**/α strain SNY87 [72] by growth on sorbose medium to select for **a** and α derivatives, as previously described [19]. PCR was used to confirm that the vector had inserted at the endogenous *SPO11* allele. To induce chromosome instability in tetraploid strains, the *SPO11* ^+^ tetraploid strain RBY18, or Δ*spo11* tetraploid strains RBY176/177, were incubated on S. cerevisiae pre-sporulation (pre-spo) medium (0.8% yeast extract, 0.3% peptone, 10% dextrose, and 2% agar) at 37 °C for 10 d. Alternatively, tetraploid strains were incubated on L-sorbose medium (0.7% yeast nitrogen base (without amino acids), 2% L-sorbose, and 2% agar) at 30 °C for 10 d. Following incubation, cells that had undergone loss of Chromosome 1 and become *gal1*− were selected by growth on 2-deoxygalactose (2-DOG) medium for 2 d, as previously described [16]. 2-DOG^+^ colonies were patched onto YPD and subsequently frozen (in a 1:1 solution of 50% glycerol and YPD). Subsequent culturing of progeny strains was kept to a minimum (less than 1 wk).

We have not attempted to reintegrate *SPO11* for three major reasons. First, the phenotype being tested is subtle; RBY18 (*SPO11* ^+^) tetraploid strains exhibited recombination events in three out of 13 progeny, while Δ*spo11* mutants exhibited no observable recombination events. Second, because we are studying a phenomenon in tetraploid strains, it is not clear how many copies of the *SPO11* gene would need to be reintegrated into the tetraploid to generate a significant difference from the mutant. And third, reconstituted strains often exhibit a range of complementation efficiencies, with multiple strains having to be analyzed to confirm restoration of the wild-type phenotype.

### PCR and flow cytometric analysis of strains.

PCR analysis of the *MTL* alleles was used as an indicator of the copy number of Chr 5 in each sample. PCR primers unique to *MTL**a**1*, *MTLα1*, Δ***a*** *1*, and Δ*α2* were used to distinguish *MTL* alleles in tetraploid cells and progeny cells derived from tetraploids. The oligonucleotides used for *MTL* analysis have been previously described [14].

To generate cells for flow cytometric analysis, test strains were grown in YPD medium at 30 °C and harvested when the OD was between 1 and 2. Samples were then prepared for analysis as previously described [16].

### SNP and CGH microarrays.

Previously, we described the development of a SNP microarray to determine genotypes at 123 SNP loci across the genome of C. albicans (Forche et al., unpublished data.). For this study the microarray was expanded to include an additional 29 SNP loci, giving a total of 152 (Table S3). Fifteen of the 29 new SNP loci were adapted from Wu et al. [73] (Table S3). Since clinical isolates were used by Wu and co-workers and not derivatives of strain SC5314 (the SNP microarray is based on SNPs from SC5314), the presence of reported SNPs was confirmed by sequencing, as described previously [25]. New primer pairs were developed to allow for the amplification of small PCR products suitable for SNP microarray analysis. Design of allele-specific oligonucleotides, probe generation, slide preparation/hybridization, data analysis, and sequence confirmation of LOH events were conducted as described elsewhere ([26]; Forche et al., unpublished data). CGH that has been adapted for C. albicans was carried out as described previously [27].

### Statistical analysis.

A two-tailed *t* test was performed to indicate if changes in karyotype were statistically significant. A *p*-value of < 0.05 in the two-tailed *t* test was interpreted as a significant difference, while *p*-values >= 0.05 were insignificant.

### Western blotting.

Cultures of strains CAY126 (Spo11-13myc), RSY84 (Kar3-13myc), and the untagged RBY1118 strain were grown to logarithmic phase in YPD medium at 30 °C and cells harvested. Whole-cell extracts from these strains were prepared by resuspending cell pellets in lysis buffer (10 mM Tris-HCl [pH 7.5], 50 mM NaCl, 1 mM dithiothreitol) containing protease inhibitors (pepstatin A, leupeptin, phenylmethyl sulfonyl chloride, and aprotinin) and lysis achieved by bead beating for 12–15 cycles (30 s vortexing following by 30–60 s on ice). An aliquot from each sample was separated by SDS-PAGE and analyzed by western blotting. The myc-tagged proteins were detected using an anti-myc antibody at 1/2,000 dilution (4a6 antibody; Millipore) followed by an anti-mouse HRP (horseradish peroxidase)-conjugated antibody at 1/1,000 dilution (Jackson Laboratories). Antibody binding was visualized using the SuperSignal West Pico Chemiluminescent Substrate (Pierce) and exposure to autoradiography film.

## Supporting Information

Figure S1 Alignment of C. albicans Spo11 with Conserved Spo11 Proteins from Other Species C. albicans Spo11 (product of *ORF19.11071*) was aligned with other fungal Spo11 sequences from S. pombe, S. cerevisiae, and K. lactis, as well as with the *Drosophila* Spo11 protein. The active site tyrosine residue is highlighted in yellow, with other highly conserved regions highlighted in blue.
(53 KB PPT)

Figure S2 Flow Cytometric Analysis of Progeny Diploid Strains Derived from Δ*spo11* Tetraploids via the Parasexual Cycle As a control, a parental diploid strain (A) and the tetraploid RBY18 strain (B) were analyzed by flow cytometry for comparison. The *x*-axis of each graph (Sytox) represents a linear scale of nuclear fluorescence, and the *y*-axis (Counts) represents a linear scale of cell number.
(32.9 MB TIF)

Figure S3 Growth Rates of Progeny Strains from Wild-Type (*SPO11* ^+^) and *Δspo11* Tetraploid Strains (A) Doubling times (DT) of each of the progeny strains during exponential growth in YPD medium at 30 °C (min).
(B) Graph indicates the correlation between increased numbers of aneuploid (trisomic) chromosomes and increased cell doubling times.
(C) Increased cell doubling times of progeny strains is dependent on the number of extra chromosomes they contain (over the normal diploid complement). The progeny strains were compared to SC5314, an **a**/α diploid control strain. The standard error is shown for the averaged data. Growth rates for progeny containing two or three trisomic chromosomes (+2 Chr or +3 Chr, respectively) differed significantly from euploid diploid strains (*p* < 0.005), using a *t*-test (two sample assuming equal variances).
(70 KB DOC)

Figure S4 CGH Analysis of Progeny Strains Derived from Wild-Type (*SPO11* ^+^) and Δ*spo11* Tetraploid Strains Plots of each chromosome are shown with the *y*-axes representing gene copy number calculated from log 2 values. Abbreviations are CSE4, centromeric DNA; MRS, major repeat sequence; transp., transposon; CARE-2, the CARE-2 repetitive element; telom., telomeric sequences.
(3.86 MB PPT)

Figure S5 Oligonucleotides Used to Amplify the *SPO11* Gene Disruption Construct (12 KB PDF)

Table S1 Comparison of CGH and SNP Data Table shows large chromosomal changes indicated by CGH and SNP analysis. These changes include chromosomal aneuploidies (trisomic chromosomes; 3×) and LOH events (whole chromosomes or partial chromosomes). This table shows the high correlation between identification of trisomic chromosomes by CGH and SNP techniques.
(23 KB XLS)

Table S2 Complementation of Spo11 Function in S. cerevisiae Meiotic Recombination SKY10 (*Δspo11*) was transformed with *ARS/CEN* plasmids carrying the indicated *SPO11* alleles. Intragenic recombination was measured as the HIS^+^ prototroph frequency (frequency × 10^3^ per viable cell) after 8 h at 30 °C in SPM medium [35]. Each value is the mean ± standard deviation of at least four experimental samples. The premeiotic prototroph frequencies have not been subtracted.
(25 KB DOC)

Table S3 Additional SNPs Used in This Study 29 additional SNPs were included in this study as listed. Each SNP has a unique identifier number (151–179) and a descriptive name (e.g., ERG7/1). The chromosomal location of each SNP in the Contig 19 database (http://www.candidagenome.org) is listed. The chromosome position is indicated by both the chromosome number and the Sfi fragment on which the SNP is found (e.g., 2U indicates the Sfi fragment U on Chromosome 2). For more details on the physical map of C. albicans go to http://albicansmap.ahc.umn.edu. Note that some SNPs are located on the same PCR product (e.g., SNPs 152, 153, and 154 are all located on the same PCR product on Chromosome 2).
(26 KB XLS)

Table S4 SNP Data for Progeny Strains Derived from the Wild-Type (*SPO11* ^+^) Tetraploid Strain (RBY18) Table shows SNP data for parental diploids (DIP), tetraploid RBY18 strain (TET), and 13 progeny (PR) strains. Strains P1 to P7 were derived from pre-spo medium, while strains S1 to S6 were derived from sorbose medium. Homozygous loci are indicated by red text (AA configuration) or pink text (BB configuration). Trisomic loci are indicated by green text (AAB configuration) or blue text (ABB configuration). Black text indicates the locus is heterozygous (AB configuration). NaN; data not applicable.
(67 KB XLS)

Table S5 SNP Data for Progeny Strains Derived from Δ*spo11* Tetraploids Grown on Pre-Spo Medium Table shows SNP data for parental diploids (DIP), tetraploid strains (RBY176/177; TET), and eight progeny (PR) strains. Strains Ps1 to Ps8 were derived from Δ*spo11* tetraploids grown on pre-spo medium. Homozygous loci are indicated by red text (AA configuration) or pink text (BB configuration). Trisomic loci are indicated by green text (AAB configuration) or blue text (ABB configuration). Black text indicates the locus is heterozygous. NaN; data not applicable.
(222 KB XLS)

Table S6 SNP Data for Progeny Strains Derived from Δ*spo11* Tetraploids Grown on Sorbose Medium Table shows SNP data for parental diploids (DIP), tetraploid strains (RBY176/177; TET), and ten progeny (PR) strains. Strains Ss1 to Ss10 were derived from Δ*spo11* tetraploids grown on sorbose medium. Homozygous loci are indicated by red text (AA configuration) or pink text (BB configuration). Trisomic loci are indicated by green text (AAB configuration) or blue text (ABB configuration). Black text indicates the locus is heterozygous. NaN; data not applicable.
(66 KB XLS)

## Abbreviations

CGH: comparative genome hybridization
DSB: double-strand break
LOH: loss of heterozygosity
*MTL*: mating-type like

## References

[pbio-0060110-b001] Kassir Y, Adir N, Boger-Nadjar E, Raviv NG, Rubin-Bejerano I (2003). Transcriptional regulation of meiosis in budding yeast. Int Rev Cytol.

[pbio-0060110-b002] Davis L, Smith GR (2001). Meiotic recombination and chromosome segregation in Schizosaccharomyces pombe. Proc Natl Acad Sci U S A.

[pbio-0060110-b003] Edmond MB, Wallace SE, McClish DK, Pfaller MA, Jones RN (1999). Nosocomial bloodstream infections in United States hospitals: a three-year analysis. Clin Infect Dis.

[pbio-0060110-b004] Ruhnke M, Maschmeyer G (2002). Management of mycoses in patients with hematologic disease and cancer: review of the literature. Eur J Med Res.

[pbio-0060110-b005] Eggimann P, Garbino J, Pittet D (2003). Epidemiology of *Candida* species infections in critically ill non-immunosuppressed patients. Lancet Infect Dis.

[pbio-0060110-b006] Gudlaugsson O, Gillespie S, Lee K, Vande Berg J, Hu J (2003). Attributable mortality of nosocomial candidemia, revisited. Clin Infect Dis.

[pbio-0060110-b007] Wey SB, Mori M, Pfaller MA, Woolson RF, Wenzel RP (1988). Hospital-acquired candidemia. The attributable mortality and excess length of stay. Arch Intern Med.

[pbio-0060110-b008] Kwon-Chung KJ, Bennett JE (1992). Medical mycology.

[pbio-0060110-b009] Hull CM, Raisner RM, Johnson AD (2000). Evidence for mating of the “asexual” yeast Candida albicans in a mammalian host. Science.

[pbio-0060110-b010] Magee BB, Magee PT (2000). Induction of mating in Candida albicans by construction of *MTLa* and *MTLalpha* strains. Science.

[pbio-0060110-b011] Lachke SA, Lockhart SR, Daniels KJ, Soll DR (2003). Skin facilitates Candida albicans mating. Infect Immun.

[pbio-0060110-b012] Dumitru R, Navarathna DH, Semighini CP, Elowsky CG, Dumitru RV (2007). In vivo and in vitro anaerobic mating in Candida albicans. Eukaryot Cell.

[pbio-0060110-b013] Odds FC, Bougnoux ME, Shaw DJ, Bain JM, Davidson AD (2007). Molecular phylogenetics of Candida albicans. Eukaryot Cell.

[pbio-0060110-b014] Miller MG, Johnson AD (2002). White-opaque switching in Candida albicans is controlled by mating-type locus homeodomain proteins and allows efficient mating. Cell.

[pbio-0060110-b015] Pujol C, Daniels KJ, Lockhart SR, Srikantha T, Radke JB (2004). The closely related species Candida albicans and Candida dubliniensis can mate. Eukaryot Cell.

[pbio-0060110-b016] Bennett RJ, Johnson AD (2003). Completion of a parasexual cycle in Candida albicans by induced chromosome loss in tetraploid strains. Embo J.

[pbio-0060110-b017] Keeney S, Lankenau D (2007). Spo11 and the formation of DNA double-strand breaks in meiosis. Recombination and meiosis.

[pbio-0060110-b018] Tzung KW, Williams RM, Scherer S, Federspiel N, Jones T (2001). Genomic evidence for a complete sexual cycle in Candida albicans. Proc Natl Acad Sci U S A.

[pbio-0060110-b019] Janbon G, Sherman F, Rustchenko E (1998). Monosomy of a specific chromosome determines L-sorbose utilization: a novel regulatory mechanism in Candida albicans. Proc Natl Acad Sci U S A.

[pbio-0060110-b020] Gorman JA, Gorman JW, Koltin Y (1992). Direct selection of galactokinase-negative mutants of Candida albicans using 2-deoxy-galactose. Curr Genet.

[pbio-0060110-b021] Nantel A, Dignard D, Bachewich C, Harcus D, Marcil A (2002). Transcription profiling of Candida albicans cells undergoing the yeast-to-hyphal transition. Mol Biol Cell.

[pbio-0060110-b022] Mitchell AP (1998). Dimorphism and virulence in Candida albicans. Curr Opin Microbiol.

[pbio-0060110-b023] Whiteway M (2000). Transcriptional control of cell type and morphogenesis in Candida albicans. Curr Opin Microbiol.

[pbio-0060110-b024] Liu H (2002). Co-regulation of pathogenesis with dimorphism and phenotypic switching in Candida albicans, a commensal and a pathogen. Int J Med Microbiol.

[pbio-0060110-b025] Forche A, Magee PT, Magee BB, May G (2004). Genome-wide single-nucleotide polymorphism map for Candida albicans. Eukaryot Cell.

[pbio-0060110-b026] Forche A, May G, Magee PT (2005). Demonstration of loss of heterozygosity by single-nucleotide polymorphism microarray analysis and alterations in strain morphology in Candida albicans strains during infection. Eukaryot Cell.

[pbio-0060110-b027] Selmecki A, Bergmann S, Berman J (2005). Comparative genome hybridization reveals widespread aneuploidy in Candida albicans laboratory strains. Mol Microbiol.

[pbio-0060110-b028] Selmecki A, Forche A, Berman J (2006). Aneuploidy and isochromosome formation in drug-resistant Candida albicans. Science.

[pbio-0060110-b029] Kraus E, Leung WY, Haber JE (2001). Break-induced replication: a review and an example in budding yeast. Proc Natl Acad Sci U S A.

[pbio-0060110-b030] Whelan WL, Soll DR (1982). Mitotic recombination in Candida albicans: recessive lethal alleles linked to a gene required for methionine biosynthesis. Mol Gen Genet.

[pbio-0060110-b031] Sarachek A, Weber DA (1986). Segregant-defective heterokaryons of Candida albicans. Curr Genet.

[pbio-0060110-b032] Diener AC, Fink GR (1996). *DLH1* is a functional Candida albicans homologue of the meiosis-specific gene *DMC1*. Genetics.

[pbio-0060110-b033] Keeney S, Giroux CN, Kleckner N (1997). Meiosis-specific DNA double-strand breaks are catalyzed by Spo11, a member of a widely conserved protein family. Cell.

[pbio-0060110-b034] Bergerat A, de Massy B, Gadelle D, Varoutas PC, Nicolas A (1997). An atypical topoisomerase II from Archaea with implications for meiotic recombination. Nature.

[pbio-0060110-b035] Diaz RL, Alcid AD, Berger JM, Keeney S (2002). Identification of residues in yeast Spo11p critical for meiotic DNA double-strand break formation. Mol Cell Biol.

[pbio-0060110-b036] Keeney S (2001). Mechanism and control of meiotic recombination initiation. Curr Top Dev Biol.

[pbio-0060110-b037] Ramesh MA, Malik SB, Logsdon JM (2005). A phylogenomic inventory of meiotic genes: evidence for sex in *Giardia* and an early eukaryotic origin of meiosis. Curr Biol.

[pbio-0060110-b038] Richard GF, Kerrest A, Lafontaine I, Dujon B (2005). Comparative genomics of hemiascomycete yeasts: genes involved in DNA replication, repair, and recombination. Mol Biol Evol.

[pbio-0060110-b039] Janbon G, Sherman F, Rustchenko E (1999). Appearance and properties of L-sorbose-utilizing mutants of Candida albicans obtained on a selective plate. Genetics.

[pbio-0060110-b040] Torres EM, Sokolsky T, Tucker CM, Chan LY, Boselli M (2007). Effects of aneuploidy on cellular physiology and cell division in haploid yeast. Science.

[pbio-0060110-b041] Gerstein AC, Chun HJ, Grant A, Otto SP (2006). Genomic convergence toward diploidy in Saccharomyces cerevisiae. PLoS Genet.

[pbio-0060110-b042] Bennett RJ, Johnson AD (2005). Mating in Candida albicans and the search for a sexual cycle. Annu Rev Microbiol.

[pbio-0060110-b043] Sarachek A, Rhoads DD, Schwarzhoff RH (1981). Hybridization of Candida albicans through fusion of protoplasts. Arch Microbiol.

[pbio-0060110-b044] Kakar SN, Partridge RM, Magee PT (1983). A genetic analysis of Candida albicans: isolation of a wide variety of auxotrophs and demonstration of linkage and complementation. Genetics.

[pbio-0060110-b045] Hilton C, Markie D, Corner B, Rikkerink E, Poulter R (1985). Heat shock induces chromosome loss in the yeast Candida albicans. Mol Gen Genet.

[pbio-0060110-b046] Poulter R, Hanrahan V (1983). Conservation of genetic linkage in nonisogenic isolates of Candida albicans. J Bacteriol.

[pbio-0060110-b047] Borner GV (2006). Balancing the checks: surveillance of chromosomal exchange during meiosis. Biochem Soc Trans.

[pbio-0060110-b048] Whitby MC (2005). Making crossovers during meiosis. Biochem Soc Trans.

[pbio-0060110-b049] Bishop DK, Park D, Xu L, Kleckner N (1992). *DMC1*: a meiosis-specific yeast homolog of *E. coli recA* required for recombination, synaptonemal complex formation, and cell cycle progression. Cell.

[pbio-0060110-b050] Shinohara A, Ogawa H, Ogawa T (1992). Rad51 protein involved in repair and recombination in S. cerevisiae is a RecA-like protein. Cell.

[pbio-0060110-b051] White TC (1997). The presence of an R467K amino acid substitution and loss of allelic variation correlate with an azole-resistant lanosterol 14alpha demethylase in Candida albicans. Antimicrob Agents Chemother.

[pbio-0060110-b052] Miyazaki Y, Geber A, Miyazaki H, Falconer D, Parkinson T (1999). Cloning, sequencing, expression, and allelic sequence diversity of *ERG3* (C-5 sterol desaturase gene) in Candida albicans. Gene.

[pbio-0060110-b053] Staib P, Kretschmar M, Nichterlein T, Hof H, Morschhauser J (2002). Host versus in vitro signals and intrastrain allelic differences in the expression of a Candida albicans virulence gene. Mol Microbiol.

[pbio-0060110-b054] Holmes AR, Tsao S, Ong SW, Lamping E, Niimi K (2006). Heterozygosity and functional allelic variation in the Candida albicans efflux pump genes *CDR1* and *CDR2*. Mol Microbiol.

[pbio-0060110-b055] Wu W, Lockhart SR, Pujol C, Srikantha T, Soll DR (2007). Heterozygosity of genes on the sex chromosome regulates Candida albicans virulence. Mol Microbiol.

[pbio-0060110-b056] Nielsen K, Heitman J (2007). Sex and virulence of human pathogenic fungi. Adv Genet.

[pbio-0060110-b057] Heitman J (2006). Sexual reproduction and the evolution of microbial pathogens. Curr Biol.

[pbio-0060110-b058] Paoletti M, Rydholm C, Schwier EU, Anderson MJ, Szakacs G (2005). Evidence for sexuality in the opportunistic fungal pathogen Aspergillus fumigatus. Curr Biol.

[pbio-0060110-b059] Goddard MR, Godfray HC, Burt A (2005). Sex increases the efficacy of natural selection in experimental yeast populations. Nature.

[pbio-0060110-b060] Grimberg B, Zeyl C (2005). The effects of sex and mutation rate on adaptation in test tubes and to mouse hosts by Saccharomyces cerevisiae. Evolution Int J Org Evolution.

[pbio-0060110-b061] Graser Y, Volovsek M, Arrington J, Schonian G, Presber W (1996). Molecular markers reveal that population structure of the human pathogen Candida albicans exhibits both clonality and recombination. Proc Natl Acad Sci U S A.

[pbio-0060110-b062] Anderson JB, Wickens C, Khan M, Cowen LE, Federspiel N (2001). Infrequent genetic exchange and recombination in the mitochondrial genome of *Candida albicans*. J Bacteriol.

[pbio-0060110-b063] Mata AL, Rosa RT, Rosa EA, Goncalves RB, Hofling JF (2000). Clonal variability among oral Candida albicans assessed by allozyme electrophoresis analysis. Oral Microbiol Immunol.

[pbio-0060110-b064] Tavanti A, Davidson AD, Fordyce MJ, Gow NA, Maiden MC (2005). Population structure and properties of Candida albicans, as determined by multilocus sequence typing. J Clin Microbiol.

[pbio-0060110-b065] Perepnikhatka V, Fischer FJ, Niimi M, Baker RA, Cannon RD (1999). Specific chromosome alterations in fluconazole-resistant mutants of Candida albicans. J Bacteriol.

[pbio-0060110-b066] Rustchenko E (2007). Chromosome instability in Candida albicans. FEMS Yeast Res.

[pbio-0060110-b067] Hughes TR, Roberts CJ, Dai H, Jones AR, Meyer MR (2000). Widespread aneuploidy revealed by DNA microarray expression profiling. Nat Genet.

[pbio-0060110-b068] Dunham MJ, Badrane H, Ferea T, Adams J, Brown PO (2002). Characteristic genome rearrangements in experimental evolution of Saccharomyces cerevisiae. Proc Natl Acad Sci U S A.

[pbio-0060110-b069] Guthrie C, Fink GR (1991). Guide to yeast genetics and molecular biology.

[pbio-0060110-b070] Fonzi WA, Irwin MY (1993). Isogenic strain construction and gene mapping in Candida albicans. Genetics.

[pbio-0060110-b071] Wilson RB, Davis D, Enloe BM, Mitchell AP (2000). A recyclable Candida albicans URA3 cassette for PCR product-directed gene disruptions. Yeast.

[pbio-0060110-b072] Noble SM, Johnson AD (2005). Strains and strategies for large-scale gene deletion studies of the diploid human fungal pathogen Candida albicans. Eukaryot Cell.

[pbio-0060110-b073] Wu W, Pujol C, Lockhart SR, Soll DR (2005). Chromosome loss followed by duplication is the major mechanism of spontaneous mating-type locus homozygosis in Candida albicans. Genetics.

