# Supplementary material for: The Parasexual Cycle in Candida albicans Provides an Alternative Pathway to Meiosis for the Formation of Recombinant Strains
Source: PLoS Biol. 2008 May 6;6(5):e110. doi: 10.1371/journal.pbio.0060110 (PMC2365976; doi:10.1371/journal.pbio.0060110)
Supplement: Supplementary file 1 [file pbio.0060110.sg005.pdf]

Spo11 KO-5’

ATGAACTATCATGAAGTGACAATTATAATTAAAAACTCACTGAACAACT
CATTAAGGAAGTTTTCCCAGTCACGACGTT

Spo11 KO-3’

TTATTTCGTATAGCTAGGCGTTCCAAGAATTTTTGAGACCATATAGTT
CACATAAGATTGTGTGGAATTGTGAGCGGATA
