# Supplementary material for: The Parasexual Cycle in Candida albicans Provides an Alternative Pathway to Meiosis for the Formation of Recombinant Strains
Source: PLoS Biol. 2008 May 6;6(5):e110. doi: 10.1371/journal.pbio.0060110 (PMC2365976; doi:10.1371/journal.pbio.0060110)
Supplement: Supplementary file 2 [file pbio.0060110.sg003.doc]

A

| **Strain** | **DT (min)** | **% increase** | **Strain** | **DT (min)** | **% increase** |
| --- | --- | --- | --- | --- | --- |
| SC5314 | 82.5 | 0 | SC5314 | 82.5 | 0 |
| RBY18 | 84.5 | 2.4 | RBY18 | 84.5 | 2.4 |
| P1 | 90.0 | 9.1 | Ps1 | 100.5 | 21.7 |
| P2 | 90.0 | 9.1 | Ps2 | 88.6 | 5 |
| P3 | 93.7 | 13.5 | Ps3 | 90 | 9.1 |
| P4 | 90.0 | 9.1 | Ps4 | 87.7 | 6.3 |
| P5 | 84.5 | 2.4 | Ps5 | 83.5 | 1.2 |
| P6 | 85.6 | 3.7 | Ps6 | 88.9 | 7.7 |
| P7 | 88.9 | 7.7 | Ps7 | 91.2 | 10.5 |
| S1 | 105.0 | 27.3 | Ps8 | 91.2 | 10.5 |
| S2 | 105.0 | 27.3 | Ss1 | 94.6 | 15.1 |
| S3 | 97.6 | 18.3 | Ss2 | 92.4 | 12 |
| S4 | 99.0 | 20.0 | Ss3 | 84.5 | 2.4 |
| S5 | 95.0 | 15.1 | Ss4 | 91.2 | 10.5 |
| S6 | 95.0 | 15.1 | Ss5 | 93.7 | 13.5 |
|  |  |  | Ss6 | 93.7 | 13.5 |
|  |  |  | Ss7 | 96.3 | 16.7 |
|  |  |  | Ss8 | 93.7 | 13.5 |
|  |  |  | Ss9 | 111.8 | 35.5 |
|  |  |  | Ss10 | 99.1 | 20.0 |

B

C

| **Diploid Progeny**  **increased DT (%)** | **Progeny (+1 Chr) increased DT (%)** | **Progeny (+2 Chr) increased DT (%)** | **Progeny (+3 Chr) increased DT (%)** |
| --- | --- | --- | --- |
| +7.4 (+/- 2.3) | +9.5 (+/- 4.2) | +16.3 (+/- 4.7) * | + 23.4 (+/- 8.6) * |
