# Supplementary material for: The Parasexual Cycle in Candida albicans Provides an Alternative Pathway to Meiosis for the Formation of Recombinant Strains
Source: PLoS Biol. 2008 May 6;6(5):e110. doi: 10.1371/journal.pbio.0060110 (PMC2365976; doi:10.1371/journal.pbio.0060110)
Supplement: Supplementary file 3 [file pbio.0060110.st002.doc]

| Plasmid-borne *SPO11* allele | Frequency of His^+^ (10^3^) |
| --- | --- |
| Vector | 0.034 ± 0.013 |
| WT *ScSPO11* | 5.8 ± 0.032 |
| *ScSPO11* (HA3His6-tagged) | 0.92 ± 0.24 |
| *CaSPO11* | 0.042 ± 0.061 |
